# Developing and Testing Strategies for Improving Cleanliness of Shared Sanitation in Low-Income Settlements of Kisumu, Kenya

**DOI:** 10.4269/ajtmh.20-1634

**Published:** 2021-10-25

**Authors:** Sheillah Simiyu, Prince Antwi-Agyei, Kwaku Adjei, Raphael Kweyu

**Affiliations:** ^1^ African Population and Health Research Center, Nairobi, Kenya;; ^2^ University of Energy and Natural Resources, Sunyani, Ghana;; ^3^ Kwame Nkrumah University of Science and Technology, Kumasi, Ghana;; ^4^ Kenyatta University, Nairobi, Kenya

## Abstract

Sharing of sanitation is common in low-income settlements in Sub-Saharan Africa. However, shared (limited) sanitation facilities have been thought to pose health risks due to poor hygiene levels. Interventions to improve user behavior and cleanliness of shared sanitation are few, both in literature and in practice. This study details the codesign and testing of strategies to improve the cleanliness of shared sanitation facilities in low-income areas of Kisumu City in Kenya. The strategies included a cleaning plan, monitoring system, and discussions among users, and were codesigned through workshops with stakeholders and group discussions with landlords and tenants. These strategies were tested in 38 compound houses through the Trials of Improved Practices approach over a 5-month period. Field staff visited the compounds, observed the cleanliness of the shared toilets, and through discussions, encouraged users to develop a formal cleaning system and a monitoring plan. The discussions built social capital and collective action and facilitated uptake of the cleaning plan with notable improvements in cleanliness of shared toilets. The results support the acceptability of shared sanitation in low-income settlements, the importance of codesigning and coproducing solutions with users, and the need to evaluate the effects of these strategies on cleanliness of shared sanitation.

## INTRODUCTION

It is estimated that between 2000 and 2017, the population using “limited” sanitation facilities increased from 5% to 8% globally.^1^ “Limited” sanitation refers to any basic sanitation facilities that are shared by two or more households. Its use is widespread in sub-Saharan Africa where the population sharing sanitation facilities doubled between 2000 and 2017.^1^ Sharing is generally considered an interim solution, especially in low-income urban areas, which lack space for individual household sanitation facilities. However, shared facilities are considered a low level of service because of health risks and human rights concerns.^1^

Health risks from sharing of sanitation facilities arise mainly because of the possibility of human contact with excreta due to poor hygiene levels. There are mixed results concerning shared sanitation in literature. A number of studies have confirmed that shared sanitation facilities are a health risk as they often contain fecal matter due to poor user behavior, lack of cleaning, and a general lack of responsibility and initiative among users to clean the facilities.^2^^–^^7^ Other studies, on the other hand, have suggested that shared toilets may not always contain fecal matter, and that they may be less contaminated, functional, and safer than individual household facilities.^8^^–^^12^

Since shared sanitation facilities are an interim solution especially for households in low-income areas, few interventions have been implemented to improve their hygiene levels. The few interventions have adopted a number of approaches, including education and the provision of cleaning materials,^13^ as well as the use of discussions to encourage users to take responsibility for cleaning and improving the quality of their facilities.^14^^,^^15^ However, these studies did not adopt a codesign approach, and only a limited number have detailed how the interventions were developed and tested prior to their implementation.^16^

Our work focuses on the management of shared sanitation facilities in the low-income settlements of Kisumu in Kenya and Kumasi in Ghana. Initial results highlighted the barriers and opportunities for improving the cleanliness of shared sanitation in the two cities.^17^^,^^18^ In this article, we detail how we developed and tested contextually relevant strategies aimed at improving the cleanliness of shared sanitation facilities. We specifically document the various strategies that were proposed and implemented by users of shared sanitation facilities in the low-income settlements of Kisumu City in Kenya. The rest of the article is structured as follows: details of the theoretical approach and a background of the study area are presented first, after which we detail the development and testing of the strategies. The stages of development and testing will be presented together with results. An overall discussion and conclusion will then follow.

### Theoretical approach.

The study was guided by the Behavior Change Wheel, a theoretical approach for designing behavior change interventions.^19^ The wheel has three rings that detail the stages of designing behavior change interventions. The inner ring requires an understanding of the existing behaviors and identification of what needs to change, the middle ring requires identification of intervention functions that are likely to initiate a change in behavior based on the inner ring, and the outer ring identifies policy categories that support the change.^19^ Our earlier manuscript detailed the results from the first stage which entailed characterizing and understanding existing behavior, as well as identification of what needs to change.^17^

For development and testing of the interventions, we adopted the Trials of Improved Practices (TIPs) approach. The TIPs approach uses a participatory methodology and aims to design feasible interventions and then test whether these interventions are acceptable.^20^ Through the TIPs approach, implementers learn from the community about what aspects of the intervention should be promoted or eliminated, the efficacy of the different approaches, and the level of impact the interventions are likely to have.^20^ Typically, TIPs is conducted after a formative research phase, it requires a small sample size (at least 20 participants), and lasts for a short time (minimum of a week) depending on the behaviors being tested.^20^ The TIPs approach enables participants to codesign and improve the interventions, which are thus more likely to be adopted by the end users.^21^^,^^22^ In this study, we used the TIPs approach to develop and test a combination of strategies that promote sustainable compound-level cleaning of shared sanitation facilities.

### Context.

This study was conducted in Kisumu, Kenya’s third largest city after Nairobi and Mombasa. The city has a population of approximately 500,000 residents as per the 2019 population and housing census.^23^ Approximately 60% of the city’s population lives in low-income settlements which are characterized by overcrowding, inadequate water and sanitation service provision, and poor housing structures.^24^^,^^25^ These low-income settlements include Nyalenda A and B, Manyatta A and B, Bandani, and Obunga. Land in these settlements is on free-hold tenure, with landowners passing on ownership from one generation to the next.^26^ Due to population growth, there has been an increase in the demand for housing, and landowners have responded by constructing housing structures for rental purposes. The structures are usually single housing units, with occupants sharing common facilities such as the yard, a water source, and toilet facilities (if available). Over time, some landowners have moved out of the settlements whereas others have continued to stay within the settlements, often residing with the tenants on the same piece of land (called a plot or compound). Compounds where the landowners have moved out are usually occupied by tenants, with some compounds having one tenant designated as a caretaker.^25^ Sanitation facilities are mainly improved pit latrines shared by an average of eight households.^4^^,^^25^ Previous studies indicated that majority of these latrines were dirty, mainly because users did not participate in their cleaning.^4^^,^^17^ The study was conducted in Nyalenda A, which is further divided into four clusters/villages, commonly called units: Western A, Kanyakwar, Dago, and Central units

## METHODS

An initial qualitative phase of the study provided insights on opportunities, barriers, and motivations for improving the hygiene conditions of the shared facilities. These have been summarized in an earlier manuscript.^17^ The results identified possible strategies for improving the cleanliness of the toilets which were improved behaviors and practices among facility users, as well as social collective efforts implemented by compound members.

### Developing the intervention strategies.

In development of the strategies, we aimed to develop and test strategies that were driven by the compound members, that could be easily implemented by community leaders and resource persons—such as Community Health Volunteers (CHVs)—and which could be sustained beyond the study period. The strategies were identified through workshops with the research team and stakeholders, and validated through group discussions with community members.

#### Workshops.

One workshop was held with the research team, and another with stakeholders. The purpose of these workshops was mainly to confirm the findings from the previous qualitative phase and to identify and prioritize possible strategies. The workshop with the research team sought to identify possible strategies for implementation at the compound-level as revealed by qualitative data. Team members read through the qualitative transcripts and identified the challenges as well as possible strategies. From this workshop, several strategies were identified, mainly:

a. Introduction of a cleaning schedule b. Hiring of community-based groups or external individuals to clean compound-level shared toilets c. Compound-level discussions with compound members d. Monitoring the usage and cleanliness of the shared toilets e. Locking toilets to keep other users away f. A “reward” system, such as praising members who voluntarily clean toilets, or warning/disciplining members who do not participate in cleaning toilets g. Awareness creation/health education and sensitization

A second workshop was held with stakeholders who had been part of the study from the beginning, and who were drawn from the public and private sectors, as well as from the community. In addition to identifying possible strategies, the workshop aimed to share results from the initial study phases with the stakeholders. After sharing results from the qualitative and quantitative phases, stakeholders were grouped in five heterogeneous teams, which consisted of representatives from the different stakeholder categories who attended the workshop. The teams were each asked to propose possible intervention strategies for improving the cleanliness of the shared sanitation facilities. Results from the five groups were consolidated and summarized into main themes representing the various proposed strategies.

Overall, stakeholders noted the inadequacy of toilets in the settlements, which led to tenants sharing sanitation facilities and others sneaking into and soiling their neighbors’ toilets. It was also highlighted that shared toilets were unclean as their users were less motivated to clean them if the toilets were constantly soiled and when cleaning was not done by all users. A number of solutions were suggested, including

• Mobilization and sensitization. Health and behavior change education, as well as communication and dialogue were recommended as strategies to increase awareness among community members on the need for improved behavior.
• Strategies at the compound-level. Stakeholders recommended strategies such as landlords taking responsibility for the provision and maintenance of sanitation facilities; landlords and tenants holding regular meetings; development of regulations and agreements among users (both landlords and tenants) on the maintenance of the sanitation facilities; locking toilets so that they are not used and soiled by people who are not involved in their upkeep; developing cleaning schedules/timetables that involve all users; and ensuring cooperation among compound members in overall management of their sanitation facilities.

Aside from these compound-level strategies, stakeholders proposed other strategies, including increasing access to reliable water supply; increasing the number of secure, private, and easy to clean sanitation facilities; and improvement of sanitation technologies such as pour-flush or sewer systems. Where pit latrines are used, it was noted that their design should allow for emptying and they should be constructed with a consideration of the topography, water table levels, and soil types found in the area. It was emphasized that county-level standards and guidelines on construction of sanitation facilities as well as the number of users per toilet were needed, which landlords would have to comply with. Finally, stakeholders proposed financing models such as including the cost of sanitation services in the cost of water and/or rent, and engaging community-based groups in provision of cleaning services.

A comparison of recommendations from both workshops showed similar proposed strategies, especially at the compound-level, with a focus on education and sensitization of community members, meetings and discussions among landlords and tenants, and regulations on use of shared toilet facilities. Strategies beyond the compound-level such as financing provisions, development of standards and guidelines, and technological improvements were noted as recommendations for further research since their testing was beyond the time frame and scope of the study.

#### Group discussions.

Group discussions were conducted with community members with the aim of selecting a combination of feasible strategies that could be tested at the compound-level. Community Health Volunteers from the four villages were engaged to identify participants from their villages. These participants had to be users of shared sanitation facilities who had been resident in the settlement for at least 3 months. To account for participants who may not attend, we aimed to recruit the maximum recommended number of 6–12 participants,^27^ and with equal representation of male and female participants. Community Health Volunteers were thus required to select three participants from each of their units, to make a total of 12 invited participants. Additionally, based on the aim of the discussions which was to provide insights into the combination of strategies, the number of discussions to be conducted was not determined a priori, but would be determined when saturation was attained during data collection.^28^

The first set of discussions was held separately with landlords and tenants, and these provided somewhat similar results. To confirm the results, two more discussions were conducted, which also gave similar results, and it was felt that there was no need for more discussions. Overall, two discussions were held with tenants and another two were held with landlords. The first discussion with landlords had a total of eight (three male and five female) participants while the second had nine (seven females and two male) participants. The first discussion with tenants had 11 (10 female and one male) participants, while the second had nine (eight female and one male) participants. These discussions were held over 2 days, with each day having separate group discussions for landlords and tenants. Each group had one research assistant who facilitated the discussion and another assistant who took notes and made observations.

Before the discussions began, participants were briefed about the study, and the research team presented the strategies for improving the hygiene conditions of shared toilets that had been identified during the workshops. The discussion then followed, and participants were asked to propose additional strategies. Tenants in the two group discussions proposed an additional strategy of provision of cleaning materials, while landlords in both discussions proposed the addition of rules and regulations. After inclusion of these strategies, participants were asked to vote for the three most important and feasible strategies. The voting was done secretly to prevent participants from influencing each other. These votes were then collected by the research team and tallied to provide the total number of votes for each strategy.

### Outcomes of the group discussions.

Table 1 summarizes the rankings from the discussions. The highest ranking strategies were cleaning plans, discussions in the compound, and monitoring. Cleaning plans and discussions in the compound both scored highly among landlords and tenants.

**Table 1:** Ranking of compound-level management strategies by tenants and landlords

Management strategies	Score					Rank
	Tenant group 1	Tenant group 2	Landlord group 1	Landlord group 2	Overall	
1. A cleaning plan	9	8	9	5	31	i
2. Discussions in the compounds	6	6	5	7	24	ii
3. Monitoring of use and cleanliness	7	4	4	7	22	iii
4. Rules and regulations	–	–	6	8	14	iv
5. Locking the toilets	2	4	3	4	13	v
6. Awareness/sensitization	3	3	5	0	11	vi
7. Groups or individuals to clean	3	0	3	3	9	vii
8. Reward system (praise, warning, or discipline)	0	1	4	1	6	viii
9. Provision of cleaning materials	3	3			6	viii

Both landlords and tenants agreed that a cleaning plan was necessary to encourage all users to take responsibility in cleaning their shared toilets.

*“ . . . A cleaning plan will help to encourage everyone to participate. All users should be assigned the responsibility of cleaning . . . ”* (male participant, group 1 discussion with landlords)

Tenants clarified that the cleaning plan would work best when the users have a common understanding, highlighting that there could be neighbors who do not know each other and do not participate in any common activities.

*“ . . . Some neighbors do not know each other, and some do not know how the toilets in their compounds are cleaned. We need to first have the discussions, to make sure that we all understand the importance of having clean toilets . . . ”* (Female participant, group 2 discussion with tenants)

As earlier stated, landlords suggested the inclusion of rules and regulations, noting that some tenants may not be cooperative, therefore cleaning of shared toilets should be part of communal living regulations. Tenants on the other hand felt that cleaning materials, including cleaning brushes and soap are an important component of having the toilets clean.

*“ . . . How shall we clean the toilets? We need to have soap, water, and a broom. Otherwise the toilets will not be clean . . . ”* (Female participant, Group 1 discussion with tenants)

### Testing the intervention strategies.

Testing of the intervention strategies was conducted from January to May 2020. The identified strategies were compared with the theoretical approach guiding the study. Previously identified strategies from the qualitative stage included use of restrictions, environmental restructuring, enablement, enhancing education and training, and improving sanitation technologies.^17^ These strategies tallied with the newly codesigned strategies, namely: development and use of cleaning plans; restricting against poor use through rules and monitoring; and discussions in the compound as a form of enablement. The proposed package of strategies entailed discussions with compound members, introduction of a cleaning plan and selection of a representative within the compound who would be in charge of monitoring the cleaning of the toilet as well as coordinating the compound meetings. Education and sensitization would be integrated into the compound discussions as part of the intervention package.

To qualify for selection to test the identified strategies, compounds needed to have at least two households and at least one toilet that was shared by the households. All the households in the compound had to be users of the toilet. The toilets needed to have a superstructure that offered privacy, a door that held in place, and a roof. Using these criteria, a CHV from each of the four units within Nyalenda A, together with the research assistants walked through the units and identified eligible compounds that met these criteria. In line with the TIPS recommendation for a small sample size, CHVs and the field staff were asked to recruit a minimum of 10 compounds from each of the units, thus making a total of 40 compounds (to also cater for losses during follow up). During the enrolment, two compounds refused to continue with the study, therefore reducing the sample size to 38 compounds that agreed to participate in testing the strategies. Community Health Volunteers and the research assistants visited the selected compounds to introduce the intervention strategies and encourage the compound members to participate, clarifying that the testing would entail attending meetings and discussions. Data collection during the first three meetings and discussions was done physically in the compounds over a period of 1.5 months. The next three follow-up sessions were done through phone calls to the compound leaders over a period of 2.5 months.

During the first visit and meeting with the compound members, CHVs introduced the research assistants, and the compound members introduced themselves. Once introductions were complete, the research assistant led the discussions by focusing on the use of their sanitation facility, prevailing cleaning practices, availability of a cleaning schedule, challenges faced in keeping the toilets clean, ways of improvement, and the importance of keeping shared sanitation facilities clean. The assistants proposed the strategies, and encouraged compound members to draft a cleaning plan (if they did not have any) and to propose a representative within the compound who would oversee the whole process. Members were encouraged to work together by mobilizing each other, identifying how best the strategies would work for them, and participating in implementing the strategies. At the end of the meeting, the members suggested a convenient date for the next meeting.

The second follow-up visit was held 1 week after the first visit. The CHV team and research assistants visited the compound members again to further discuss their experiences with trying out the strategies. During the meeting, compound members reported challenges encountered, progress made, and suggestions for improvement. Research assistants noted those cases where progress had been made and encouraged the members to work together in improving the cleanliness of the toilets. Where little or no progress had been made, the research assistants still encouraged the compound members to work together and try out the proposed strategies. Again, the members proposed the most convenient date for the next visit.

The third follow-up visit and discussion was done approximately 2 weeks after the second visit, to allow compound members to continue with the strategies without solely relying on the research team. This visit followed a similar format as the second visit, focusing on progress made, challenges experienced, and any outcomes from the discussions and strategies.

During these three visits, assistants collected quantitative information such as the number of compound members who attended, the type of toilets, and the number of cubicles being shared. They also collected qualitative data including the strategies that had been implemented by the compound members, challenges faced, and benefits experienced. Where possible, the assistants observed the cleanliness of the toilets. Compound meetings were only held if a minimum of 50% of all compound members who used the toilet were available. If the members were not available or the numbers were not enough, the meetings were postponed to a time that was convenient for all members.

Three more follow-up sessions were conducted remotely through phone calls due to restrictions in fieldwork activities as a result of the COVID-19 pandemic. The fourth and fifth follow-up visits were held approximately 3 weeks after the preceding visits while the sixth and final follow-up was held 1 month later. Data was collected through phone calls to the compound representatives, and to at least two other compound members. Discussions during the phone calls focused on the progress made and challenges faced.

### Ethical procedures.

Ethical approval for the study was obtained from the ethics committee of the Great Lakes University of Kisumu GLUK (Ref: GREC/001/285/2018). A research permit was further obtained from the Kenya National Commission of science and Technology (Ref: NACOSTI/P/18/5546/24979) and from the Kisumu County government. Permission to conduct the study was sought from administrative heads at the local level in the study area.

During the group discussions, field assistants detailed the purpose of the study, progress and findings from previous phases, the purpose of the group discussions, duration of the discussions, actions that would be taken to ensure anonymity and confidentiality such as not using names, and the next steps after the discussions. They were also informed that they were free not to continue with the discussions if they chose to. Discussions began after all questions from the participants had been answered satisfactorily, and participants had given their signed consent to participate in the discussion.

During the first household visits, field assistants provided detailed information sheets to the participants. The assistants explained the details of the information sheet, including the purpose of the study, reasons why the compounds were selected, the study procedures including community dissemination plans, duration of the discussions, expected requirements from the participants, steps to ensure confidentiality, how the data would be used, and contact information of the researchers. They were allowed to ask questions after which they were given a copy of the information sheet to keep. The participants signed two copies of the consent form in the presence of the CHVs. They retained one copy of the consent form, and another was kept by the study team.

### Data management and analysis.

Quantitative data from the compound visits was analyzed through descriptive statistics such as means, frequencies, and percentages. Qualitative findings from the group discussions were analyzed the matically using already predefined themes, that is, the intervention strategies. Quantitative and qualitative data were combined during analysis and presented together to provide meaningful explanations for the uptake of the strategies.

## Results

The 38 selected plots began the compound discussions but six compounds dropped out during the second follow-up visit due to the unavailability of members or the contact persons (*N* = 2), requests for compensation for attending meetings (*N* = 2), difficult/uncooperative members (*N* = 1), and filled-up pit latrines which made it difficult to implement the strategies (*N* = 1). Three other compounds dropped out during the third visit because they were not willing to participate in the strategies (*N* = 1), or due to unwilling or uncooperative members (*N* = 2). A total of 29 compounds completed all the follow-up visits (Table 2).

**Table 2:** Descriptive summary of the compounds

Variable (*N* = 38 unless specified)	Frequency (%)
Type of compound	
Tenant only	17 (45)
Resident landowner	16 (42)
Compounds with a caretaker	5 (13)
Type of toilet	
Pour flush to septic tank	3 (8)
Pit latrine with slab	35 (92)
Average number of households in the compound sharing the toilets	6 (Range: 2–12)
Number of cubicles shared	2 (Range: 1–4)
Attendance of meetings	
First visit (*N* = 38)	Average: 4.5 (Range: 2–10)
Second visit (*N* = 32)	Average: 4.4 (Range: 2–8)
Third visit (*N* = 29)	Average: 4 (Range: 2–8)

Compounds had an average of six households, and most (45%) had tenants as the main occupants. Over 90% of the toilets were pit latrines which had one to four cubicles that were shared by the households (Table 2).

### Strategy 1: Uptake and attendance of compound-level meetings and discussions.

Most members of the compounds attended the three group meetings and discussions. One compound in particular recorded 100% attendance in all the meetings, while all the other compounds had a minimum of half the number of households attending. As noted in Table 2, whereas the average number of households in the compounds was six, averagely five households were present during the meetings. In some compounds (*N* = 12), we encountered new compound members who had not attended the first meeting participating in the second and third follow-up meetings. This increase was because compound members and the compound representatives shared the information with their neighbors, encouraging them to attend the meetings. A tenant in one of the compounds gave reasons why she encouraged her neighbors to attend the meetings . . .

*“because cleanliness of the toilet affects us all, and we need to discuss together as a team”* (Female tenant, Kanyakwar unit)

In addition, members selected a convenient time and date, and in doing so, they prioritized the meetings, as noted by a participant who confirmed that,

*“We know that Sunday is a day for the meeting and discussions”* (Female tenant, Kanyakwar unit)

Furthermore, compound members often reported that they held subsequent meetings (in addition to the meetings we held with them), where they further discussed implementation of the strategies and other matters affecting them. Members who did not attend the discussions were away due to work, travel, or social functions. Whereas both men and women attended the discussions, women were the majority, partly because they were always at home, or were usually involved in cleaning of the toilets.

### Strategy 2: Uptake of the cleaning plan.

During the first visit, almost all the compounds reported that they did not have any form of cleaning plan. Similar to results from the earlier qualitative phase,^17^ toilets were cleaned by any household on a voluntary basis, or they were cleaned by individuals from specific households (e.g., women who had children). A few (13%) compounds admitted that all households in the compound were involved in cleaning, although without any form of schedule/plan.

By the end of our visits, approximately 66% of the compounds had adopted a cleaning plan (Table 3). This plan was either a written schedule or an informal cleaning plan where households organized themselves according to the order of houses or days of the week. Where households had a written plan, the schedule was often pasted on the door of the toilet or written on a paper and kept by the compound representative. This plan had the names of individuals from all the households or their house numbers. According to these plans, toilets were cleaned on a daily basis, and members often collectively selected their preferred cleaning days. In one compound, households had a weekly cleaning plan before our visits, which they opted to continue with.

**Table 3:** Cleaning practices before and after the intervention strategies

Variable	Before (*N* = 38) Frequency (%)	After (*N* = 32) Frequency (%)
Availability of a cleaning plan/schedule		
Yes*	1 (2.6)	21 (66)
No/No need	37 (97.4)	11 (34)
Cleaning arrangements		
No cleaning	3 (8)	2 (6)
Cleaning done by specific households	3 (8)	2 (6)
Voluntary cleaning	28 (74)	1 (3)
Organized cleaning	4 (10)	27 (84)
Who is involved in cleaning/management		
All households	5 (13)	15 (47)
Any household	25 (66)	1 (3)
No cleaning	3 (8)	2 (6)
Specific households	5 (13)	14 (44)

* After the visits, compounds had a cleaning plan, even when it was not always written. The “NO” also includes compounds who had some order in cleaning the toilet, but they felt that there was no need for a cleaning plan.

Informal plans were agreed upon by members, after they discussed and organized themselves. For example, participants in several compounds reported making verbal agreements during the meetings and assigning each household a specific day to clean the toilet. Two compounds in particular felt that they did not need to write down a formal plan and have it pasted on the toilet because it would be plucked out. In other compounds, especially those with few households, members reported *no need* for a formally written cleaning plan. Households from such compounds organized themselves and often assigned households to specific days of the week to clean the toilets. Such households frequently reported that they were few in number and each household knew when they were to clean the toilet.

Adoption of the discussions and the cleaning plan led to over 80% of the compounds implementing some form of organized cleaning, compared with the pre-intervention stage where households volunteered to clean toilets (74%). Notably, 47% of compounds had all households involved in the overall management of their sanitation facility. With regards to gender, men were directly involved in cleaning in most of the compounds. When men were not directly involved, compound members agreed to have women clean the toilets while men purchased cleaning materials. In such arrangements, although all households were involved in the overall management of the sanitation facilities, only specific households (especially those where women were often at home) cleaned the toilets, hence the increase from 13% in the pre-intervention stage, to 44% of cases where specific households were involved in cleaning. Before implementation of the intervention strategies, the specific households that were involved in cleaning were those with younger children, with little or no participation by other households.

The plan to have men purchase cleaning materials and have women clean the toilets was acceptable to compound members, as it was noted that some women were not comfortable with asking men to clean the toilets. In one compound for example, a female tenant confessed that:

*“I am uncomfortable asking him* [a male neighbor] *to clean the toilet”* (Female tenant, Kanyakwar unit)

Another female tenant in the same unit felt that:

*“It is women who need to clean the toilets, and not men”* (Female tenant, Kanyakwar unit)

In such compounds, men were involved in purchasing the cleaning items while women cleaned the toilets. Men were also exempted from cleaning in cases where they were unmarried and/or not always at home. In instances where individuals were not available to clean the toilets, they agreed to exchange their cleaning days with their neighbors, or they asked their neighbors to clean the toilets on their behalf. In one compound for example, a man opted to pay his neighbor to clean the toilet.

Compounds experienced various challenges with the cleaning schedule, mainly its getting torn or plucked from the toilet door (*N* = 3). Lack of materials to paste the written schedule on the toilet door was also a common challenge. Other challenges arose from the movement of tenants out of the compounds, or their movement to rural areas especially during the COVID-19 pandemic. Consequently, the available households cleaned the toilets on the days when their neighbors were to clean, or they had to rewrite the cleaning plan and only involve the available households.

### Strategy 3: Monitoring and taking responsibility.

Before the introduction of the intervention strategies, none of the tenant-only compounds had any individual who was responsible for overseeing cleanliness of the sanitation facilities. Landowners and caretakers often took on this responsibility in compounds where they resided mainly because of their status as landowners or caretakers in the compound. Compound members selected the caretaker (8%), landowner (34%), or one of the tenants (42%) to be responsible for monitoring the progress of the strategies that were being implemented in their compounds. In compounds where the landowners were resident, members selected them to oversee and be responsible for the initiatives, while in compounds without a resident landowner, members selected the caretaker or one of the tenants. Besides being our contact persons within the compound, the selected individuals also followed up with members and reminded them of meetings, they organized the compound members and drafted the cleaning plan, they followed up on any individuals who missed the discussions, and reminded members to clean the toilets. In a handful of the compounds (16%), the members felt that they did not need anyone to act as a monitor, since “*they were all adults and could organize themselves.*”

### Other strategies that were implemented.

In addition to the proposed strategies, compounds implemented additional strategies, which included individual or collective contribution of resources for purchase of cleaning materials and agents (*N* = 14); purchase of a padlock and locking of the toilets (*N* = 9); cleaning and slashing grass next to the toilet (*N* = 1); emptying of filled up pit latrines (*N* = 1); accompanying young children to the toilet to avoid misuse (*N* = 1); installing handwashing containers (*N* = 5); cleaning the bathrooms in addition to the toilets (*N* = 4); and instituting rules for use of the toilet (such as not dumping diapers in toilets, not allowing households from other compounds to use the toilet, warning and evicting members who do not participate in cleaning) (*N* = 4).

### Reported benefits.

Compound members reported various benefits as a result of the strategies. The most evident benefits were greater cooperation/teamwork among members, unity, and improved relationships among households. For instance, compound members in all the units reported that “*they were able to share and solve their problems*” or that “*they became united.*” Improved relationships contributed to the success of the strategies, as noted by one landlord respondent:

*“The good relationship among the members enhanced the implementation of the cleaning plan”* (Landlord, Central Unit)

A tenant from another compound also confirmed that the improved relationship led to households reminding each other to clean the toilets.

*“Households remind each other when it was their turn to clean”* (Female tenant, Western A unit)

The improved relationships made it easier for compound members to organize themselves and participate in other compound-level activities such as cleaning the compound and barring outsiders from sneaking into and using their toilets. Individuals who previously cleaned the toilets voluntarily without help from their neighbors reported satisfaction and relief from everyday cleaning and from division of work among all households.

Participants also reported that their toilets were cleaner because they were locked to prevent use by non-compound members who soiled them, and due to regular cleaning. One landlord for example, proudly stated that

*“My toilet is the cleanest in the area”* (Landlord, Kanyakwar unit).

Participants also reported that the toilets were cleaner because of improved behavior. Households used the toilets responsibly since they understood the effort involved in cleaning the toilet. Compounds that collectively bought cleaning soap expressed satisfaction by stating that the odor from the toilet had reduced. The cleanliness of the toilets was confirmed by observations from the research assistants, as some compounds (*N* = 10) showed improvement in the hygiene levels of their toilets from a soiled and smelly state during the first visit, to the reduction or total elimination of the odor and fecal matter around the toilet by the third visit. In terms of health benefits, one respondent reported that an infection that was suspected to have arisen from using a dirty toilet had cleared.

*“Since we started cleaning the toilet, the infection has disappeared, and this has encouraged us to continue cleaning the toilet”* (Female Tenant, Central Unit)

Other reported benefits included improved relationships between the CHVs and the community members, as well as the sharing of experiences with neighboring compounds and encouraging them to keep their toilets clean.

## DISCUSSION

Using a participatory approach based on the Behavior Change Wheel, this study details the development and testing of compound-level strategies to improve the cleanliness of shared sanitation facilities in low-income settlements of Kisumu City in Kenya. The strategies were developed through a series of workshops and group discussions with stakeholders and community members. The intervention package included activation of discussions within the compounds, development of a cleaning plan, and instituting a monitoring system. These strategies were tested in selected compounds through a series of follow-up visits led by the research team and CHVs. Uptake of the strategies showed their potential for implementation, as the discussions enabled compound members to formulate a cleaning plan and select individuals to oversee their initiatives. Additionally, the strategies led to compound members adopting other strategies collectively, which together contributed to their involvement and participation.

The development phase of the intervention entailed several workshops and group discussions. These were necessary to validate the information obtained from one data source and served as a form of internal validation. Results from the workshops spoke to similar intervention strategies, which were then confirmed by group discussions. The policy level strategies that were proposed during the stakeholder’s workshop are worth noting as they speak to the policy-level strategies in the theory used. For example, in this study, policy-level strategies proposed during earlier qualitative research indicated the need for guidelines, regulations, and environmental planning^17^ and these needs were also identified by stakeholders at the workshops. At the compound-level, landowners suggested the inclusion of rules and regulations around shared sanitation facilities, while tenants suggested provision of materials for cleaning the facilities. This divergence between landowners and tenants speaks to the different roles played by the two groups in sanitation, as landlords are owners and may wish to minimize additional costs to their sanitation facilities, whereas tenants who are everyday users of the shared toilets involved in their day-to-day cleaning would prioritize maintenance of the facilities.^29^^,^^30^ The most important aspect highlighted in this study, however, is the importance of involving end users throughout the research process, including during the development and testing of interventions to improve user experience. Landlords for example, invest in sanitation, while tenants are more transient and may not be deeply invested in the long-term. However, both groups are critical in coproducing solutions and improving the cleanliness of shared sanitation facilities.

Regarding the intervention strategies developed, we realize that some of the tested strategies are in line with the Ostrom principles mentioned in previous studies related to shared sanitation^4^^,^^31^ however, the study limited itself in testing the strategies identified from the group discussions and the workshops. More specifically, it was noted that compounds benefited from discussions among their members. A similar approach has been tried in Uganda, where discussions improved the behavior of individuals in terms of cleaning their shared toilets.^14^ These discussions also improved and strengthened relationships among compound members, promoted unity, trust and cooperation among them, and enabled collective action, above and beyond the strategies that had been proposed. These results corroborate previous studies that social capital and collective action are necessary and important in the management of shared sanitation facilities, and that social networks and relationships play a significant role in the success of collective actions.^31^^,^^32^ The importance of social capital and collective action was demonstrated through the adoption of the cleaning plan. After the compound discussions, members trusted each other and participated in cleaning, sometimes without a formally written schedule while others contributed resources, and some households cleaned for their neighbors. Other equally important social dynamics include gender and social/cultural norms. Our study contributes to previous results which showed that women are involved in or considered to be responsible for everyday sanitation aspects.^18^^,^^32^^–^^34^ A social/cultural bias was evident in our study, as men were excused from actual cleaning, and were instead allowed to participate in other ways, such as purchasing cleaning materials. Notably, not all men were excused, as others were willing to, and actually participated in the cleaning as well as other activities. These results point to the need for understanding such dynamics and the motivations that influence participation of all users to avoid a state of collective action failure.^35^

It is important to note that clean shared toilets are acceptable to users in low-income areas. Although studies have highlighted decreasing satisfaction among users with shared sanitation facilities,^36^^–^^38^ this dissatisfaction may be due to lack of cooperation and unclean toilets. It is therefore possible that levels of dissatisfaction may reduce when there is collective action and users have clean toilets. This assertion is supported by recent studies indicating that cleanliness, privacy, and safety/security are attributes that make shared sanitation facilities acceptable,^39^ and which may eventually lead to other benefits such as mental well-being.^40^ Our results therefore support the importance of focusing on behavior of users of shared sanitation facilities especially in low-income areas.

In terms of designing an intervention package, results indicate that strategies to improve the cleanliness and management of shared sanitation facilities need to be considered as a collective package, and not individually. In our case, strategies entailed joint discussions that incorporated education and awareness, development of a management system through organized cleaning plans, and a form of monitoring. The discussions were necessary to develop an agreed upon cleaning plan, and to select individuals who would monitor the cleanliness. Notably, these cleaning plans were modified to suit the users. For example, some preferred a daily schedule, whereas others preferred a weekly schedule. The form of monitoring was also agreed upon by the compound members, with some compounds choosing to have an individual assigned as the leader, and others opting to take up personal responsibility. We also note that each of the intervention strategies may need to be modified to increase their suitability for uptake. For example, although many households adopted some form of a cleaning schedule, having the cleaning plan stuck on the door may not be sustainable, and other approaches, like the modified form of a cleaning schedule implemented in Zambia, may have to be considered.^15^ Guided by the theory, we note that intervention strategies should not only include collective action, but should also have both personal and collective benefits since shared toilets are akin to public goods.^41^^,^^42^ Personal benefits may for example be realized through improved well-being, and collective benefits through clean shared toilets. Actions are necessary to ensure that collective action failure does not occur. In informal settlements, such failures may be perpetuated by the movement of members out of the compound, or a lack of trust among compound members.

This study was not without limitations. It was not a cause and effect study, and we certainly did not aim to show the effect of the strategies on the cleanliness of shared toilets or specific health outcomes. While we aimed to develop and test implementable strategies, we recommend that implementation of these strategies may best be evaluated by quantitative approaches which examine the effects of the strategies on measurable outcomes. Secondly, data collection during the last three stages was done through phone calls, and although we were not able to ascertain the validity of the results due to movement restrictions, we confirmed the responses by making phone calls to other compound members. It is possible that the cleanliness of the reported sanitation facilities was a subjective observation, a limitation that can be addressed in further studies. Finally, the study was limited by the available sanitation technologies which in turn affected any interventions to clean the toilets (e.g., pit latrines that filled up). This challenge may need to be addressed at a wider level, such as the city level, by the planning, environment and public health departments to determine appropriate sanitation interventions in low-income settlements.

## CONCLUSION

Although shared sanitation facilities are perceived as posing health risks to users, they are a common phenomenon in low-income settlements where residents may not be able to afford individual household sanitation facilities. This study details the development and testing of strategies to improve the cleanliness of shared sanitation facilities. Results highlight that if users of shared sanitation facilities are empowered, they are able to collaboratively work together to improve the cleanliness of their facilities, and thus reduce their health risks. Social capital and collective action are important prerequisites for behavior change and their development should be examined within each context. Possible interventions for large-scale implementation should incorporate aspects that enable users to build social capital and improve community behavior, while stakeholders such as local government should define minimum criteria and guidelines for provision of sanitation facilities.

## References

[1] UNICEF and World Health Organization (WHO), 2019. *Progress on Household Drinking Water, Sanitation and Hygiene 2000–2017*. Special Focus on Inequalities. New York, NY: United Nations Children's Fund (UNICEF) and World Health Organization.

[2] Heijnen M Routray P Torondel B Clasen T , 2015. Shared sanitation versus individual household latrines in urban slums: a cross-sectional study in Orissa, India. Am J Trop Med Hyg 93: 263–268. 26123953 10.4269/ajtmh.14-0812 PMC4530745

[3] Heijnen M Cumming O Peletz R Chan GKS Brown J Baker K Clasen T , 2014. Shared sanitation versus individual household latrines: a systematic review of health outcomes. PLoS One 9: e93300. 24743336 10.1371/journal.pone.0093300 PMC3990518

[4] Simiyu S Swilling M Cairncross S Rheingans R , 2017. Determinants of quality of shared sanitation facilities in informal settlements: case study of Kisumu, Kenya. BMC Public Health 17: 68. 28077103 10.1186/s12889-016-4009-6 PMC5225536

[5] Tumwebaze IK , 2013. Prevalence and determinants of the cleanliness of shared toilets in Kampala slums, Uganda. J Public Health (Bangkok) 22: 33–39.

[6] Baker KK 2016. Sanitation and hygiene-specific risk factors for moderate-to-severe Diarrhea in young children in the global enteric multicenter study, 2007–2011: case-control study. PLoS Med 13: 2007–2011. 10.1371/journal.pmed.1002010 PMC4854459 27138888

[7] Aluko OO Oloruntoba EO Chukwunenye UA Henry EU Ojogun E , 2018. The dynamics and determinants of household shared sanitation cleanliness in a heterogeneous urban settlement in southwest Nigeria. Public Health 165: 125–135. 30390425 10.1016/j.puhe.2018.09.013

[8] Exley JLR Liseka B Cumming O Ensink JHJ , 2015. The sanitation ladder, what constitutes an improved form of sanitation? Environ Sci Technol 49: 1086–1094. 25513885 10.1021/es503945x

[9] Jenkins MW Cumming O Scott B Cairncross S , 2014. Beyond “improved” towards “safe and sustainable” urban sanitation: assessing the design, management and functionality of sanitation in poor communities of Dar es Salaam, Tanzania. J Water Sanit Hyg Dev 4: 131.

[10] Montgomery M Desai MM Elimelech M , 2010. Comparing the effectiveness of shared versus private latrines in preventing trachoma in rural Tanzania. Am J Trop Med Hyg 82: 693–695. 20348521 10.4269/ajtmh.2010.09-0540 PMC2844581

[11] Berendes DM 2018. Urban sanitation coverage and environmental fecal contamination: links between the household and public environments of Accra, Ghana. PLoS One 13: 1–19. 10.1371/journal.pone.0199304 PMC6029754 29969466

[12] Massa K Kilamile F Safari E Seleman A Mwakitalima A Balengayabo JG Kassile T Mangesho PE Mubyazi GM , 2017. Contributing to the debate on categorising shared sanitation facilities as “unimproved”: an account based on field researchers’ observations and householders’ opinions in three regions, Tanzania. PLoS One 12: 1–23. 10.1371/journal.pone.0185875 PMC5673168 29107947

[13] Alam MU 2017. Behaviour change intervention to improve shared toilet maintenance and cleanliness in urban slums of Dhaka: a cluster-randomised controlled trial. Trop Med Int Health 22: 1000–1011. 28556458 10.1111/tmi.12902

[14] Tumwebaze IK Mosler HJ , 2015. Effectiveness of group discussions and commitment in improving cleaning behaviour of shared sanitation users in Kampala, Uganda slums. Soc Sci Med 147: 72–79. 26547047 10.1016/j.socscimed.2015.10.059

[15] Tidwell JB Chipungu J Bosomprah S Aunger R Curtis V Chilengi R , 2019. Effect of a behaviour change intervention on the quality of peri-urban sanitation in Lusaka, Zambia: a randomised controlled trial. Lancet Planet Health 3: e187–e196. 31029230 10.1016/S2542-5196(19)30036-1

[16] Tidwell JB Chipungu J Chilengi R Curtis V Aunger R , 2019. Using a theory-driven creative process to design a peri-urban on-site sanitation quality improvement intervention. BMC Public Health 19: 1–11. 31088432 10.1186/s12889-019-6898-7 PMC6518808

[17] Simiyu S Kweyu R Antwi-Agyei P Adjei K , 2020. Barriers and opportunities for cleanliness of shared sanitation facilities in low-income settlements in Kenya. BMC Public Health 20: 1–12. 33129296 10.1186/s12889-020-09768-1 PMC7603673

[18] Antwi-agyei P Dwumfour-asare B Adjei KA Kweyu R Simiyu S , 2020. Understanding the barriers and opportunities for effective management of shared sanitation in low-income settlements—the case of Kumasi, Ghana. Int J Environ Res Public Health 17: 4528. 10.3390/ijerph17124528 PMC7345014 32586062

[19] Michie S Van Stralen MM West R , 2011. The behaviour change wheel: a new method for characterising and designing behaviour change interventions. Implement Sci 6: 42. 21513547 10.1186/1748-5908-6-42 PMC3096582

[20] Manoff Group , 2005. *Trials of Improved Practices (TIPs)*: *Giving Participants a Voice in Program Design*. Available at: https://www.manoffgroup.com/wp-content/uploads/summarytips.pdf%0Ahttp://www.manoffgroup.com/resources/summarytips.pdf.

[21] Harvey SA Olórtegui MP Leontsini E Asayag CR Scott K Winch PJ , 2013. Trials of improved practices (TIPs): a strategy for making long-lasting nets last longer? Am J Trop Med Hyg 88: 1109–1115. 23530074 10.4269/ajtmh.12-0641 PMC3752810

[22] Sanders EB Stappers PJ , 2008. Co-creation and the new landscapes of design. CoDesign 4: 5–18.

[23] KNBS , 2019. *Kenya Population and Housing Census Volume IV*. Nairobi, Kenya: KNBS.

[24] Habitat, 2005. *Situation Analysis of Informal Settlements in Kisumu*. Nairobi, Kenya: UN-HABITAT.

[25] Simiyu S Cairncross S Swilling M , 2019. Understanding living conditions and deprivation in informal settlements of Kisumu, Kenya. Urban Forum 30: 223–241.

[26] Huchzermeyer M , 2009. Enumeration as a grassroot tool towards securing tenure in slums: insights from Kisumu, Kenya. Urban Forum 20: 271–292.

[27] Johnson RB Christensen L , 2014. *Educational Research*: *Quantitative, Qualitative, and Mixed Approaches*, 5th edition. Los Angeles, CA: Sage Publications.

[28] Hennink MM Kaiser BN Weber MB , 2019. What influences saturation? Estimating sample sizes in focus group research. Qual Health Res 29: 1483–1496. 30628545 10.1177/1049732318821692 PMC6635912

[29] Simiyu S Swilling M Cairncross S , 2017. Decision-making on shared sanitation in the informal settlements of Kisumu, Kenya. Int J Environ Health Res 27: 377–393. 28705015 10.1080/09603123.2017.1350261

[30] Antwi-Agyei P Monney I Dwumfour-Asare B Cavill S , 2019. Toilets for tenants: a cooperative approach to sanitation bye-law enforcement in Ga West, Accra. Environ Urban 31: 293–308.

[31] Chipungu J Tidwell JB Chilengi R Curtis V Aunger R , 2018. The social dynamics around shared sanitation in an informal settlement of Lusaka, Zambia. J Water Sanit Hyg Dev 9: 102–110.

[32] Shiras T Cumming O Brown J Muneme B Nala R Dreibelbis R , 2018. Shared sanitation management and the role of social capital: findings from an urban sanitation intervention in Maputo, Mozambique. Int J Environ Res Public Health 15: 2222. 10.3390/ijerph15102222 PMC6210686 30314299

[33] Kwiringira J , 2017. Barriers to Shared Sanitation Cleaning and Maintenance in Kampala Slums, Uganda. 40th WEDC International Conference, Loughborough, 1–6.

[34] Kwiringira J Atekyereza P Niwagaba C Günther I , 2014. Gender variations in access, choice to use and cleaning of shared latrines: experiences from Kampala slums, Uganda. BMC Public Health 14: 1180. 25407788 10.1186/1471-2458-14-1180 PMC4247598

[35] Tumwebaze IK Mosler HJ , 2014. Why clean the toilet if others don’t? Using a social dilemma approach to understand users of shared toilets’ collective cleaning behaviour in urban slums: a review. J Water Sanit Hyg Dev 4: 359–370.

[36] Günther I Horst A Luthi C Mosler HJ Niwagaba CB Tumwebaze IK , 2011. *Where Do Kampala’s Poor “go”? Urban Sanitation Conditions in Kampala’s Low-Income Areas*. Zürich, Switzerland: Swiss Federal Institute of Technology.

[37] Nelson KB Karver J Kullman C Graham JP , 2014. User perceptions of shared sanitation among rural households in Indonesia and Bangladesh. PLoS One 9: e103886. 25090096 10.1371/journal.pone.0103886 PMC4121202

[38] Tumwebaze IK Orach CG Niwagaba C Luthi C Mosler HJ , 2013. Sanitation facilities in Kampala slums, Uganda: users’ satisfaction and determinant factors. Int J Environ Health Res 23: 191–204. 22873693 10.1080/09603123.2012.713095

[39] Schelbert V 2020. When is shared sanitation acceptable in low-income urban settlements? A user perspective on shared sanitation quality in Kumasi, Kisumu and Dhaka. J Water Sanit Hyg Dev 10: 959–968.

[40] Shiras T Cumming O Brown J Muneme B Nala R Dreibelbis R , 2018. Shared latrines in Maputo, Mozambique: exploring emotional well-being and psychosocial stress. BMC Int Health Hum Rights 18: 1–12. 30045729 10.1186/s12914-018-0169-z PMC6060455

[41] McGranahan G , 2015. Realizing the right to sanitation in deprived urban communities: meeting the challenges of collective action, coproduction, affordability, and housing. World Dev 68: 242–253.

[42] McGranahan G Mitlin D , 2016. Learning from sustained success: how community-driven initiatives to improve urban sanitation can meet the challenges. World Dev 87: 307–317.

